# A New Cutting Device Design to Study the Orthogonal Cutting of CFRP Laminates at Different Cutting Speeds

**DOI:** 10.3390/ma12244074

**Published:** 2019-12-06

**Authors:** Víctor Criado, Norberto Feito, José Luis Cantero Guisández, José Díaz-Álvarez

**Affiliations:** 1 Department of Mechanical Engineering, Universidad Carlos III de Madrid, Avda. de la Universidad 30, Leganés, 28911 Madrid, Spain; vcriado@ing.uc3m.es (V.C.); jcantero@ing.uc3m.es (J.L.C.G.); 2 Centre of Research in Mechanical Engineering—CIIM, Department of Mechanical and Materials Engineering, Universitat Politècnica de València, Camino de Vera, s/n, 46022 Valencia, Spain; norfeisa@upvnet.upv.es

**Keywords:** orthogonal machining, CFRP, linear cutting movement, high cutting speed, experimental method

## Abstract

Carbon Fiber-reinforced plastics (CFRPs) are widely used in the aerospace industry due to their highly mechanical properties and low density. Most of these materials are used in high-risk structures, where the damage caused by machining must be controlled and minimized. The optimization of these processes is still a challenge in the industry. In this work, a special cutting device, which allows for orthogonal cutting tests, with a linear displacement at a wide range of constant cutting speeds, has been developed by the authors. This paper describes the developed cutting device and its application to analyze the influence of tool geometry and cutting parameters on the material damage caused by the orthogonal cutting of a thick multidirectional CFRP laminate. The results show that a more robust geometry (higher cutting edge radius and lower rake angle) and higher feed cause an increase in the thrust force of a cutting tool, causing burrs and delamination damage. By reducing the cutting speed, the components with a higher machining force were also observed to have less surface integrity control.

## 1. Introduction

Carbon Fiber Reinforced Polymer laminates (CFRPs) have been widely used in recent years in different industrial applications, such as the aerospace or automotive industries, due to their excellent mechanical properties and low density [1].

CFRPs are classified as difficult to cut due to the presence of hard fibers, which can cause mechanical wear to cutting tools and their inhomogeneity and their anisotropic material properties [2,3]. At the same time, the polymeric matrix can be thermally damaged due to the high temperatures reached during machining, which causes chemical and irreversible mechanical degradation of the polymer matrix [4]. All these difficulties encountered in the machining of CFRP laminates have generated a high interest in the investigation of cutting parameters, cutting conditions and tool selection. A proper selection of these conditions can considerably reduce the damage caused by workpieces and increases the performance of cutting tools.

CFRPs can be manufactured following different manufacturing methods, producing a near-net shape. However, due to the demanding tolerances, it is necessary, in most cases, to eliminate the excess material using finishing cutting processes. In addition, it is also often necessary to make holes to join different parts of huge structures, and drilling is therefore also one of the most common operations associated with these materials [5,6]. Mechanical damage, such as delamination or thermal damage to the matrix, may occur during the machining process, leading to the rejection of the produced component. Hence, the most frequent experimental studies focus on postprocessing, such as milling [7] or drilling [8], where the orientation of the fibers with respect to the direction of the workpiece-tool movement varies continuously, and it is extremely difficult to determine the influence of the fiber orientation in those cases.

The orientation of the fibers in the stacking sequence of the laminate during the material removal process has attracted great attention. It has been studied in several works on orthogonal cutting, in which the cutting edge is perpendicular to the cutting movement direction [9,10,11,12]. This process is very useful, because it simplifies the working conditions. Hence, it is easier to analyze the effect of the fiber orientation on the damage found on the machined surface of a workpiece and on tool wear [10].

A number of authors have used conventional experimental systems to perform cutting tests with a linear displacement [13,14,15,16], but these tests are very limited in terms of the maximum possible cutting speeds (values of around 5 m/min). Zitoune et al. [13] showed that the fiber orientation has a large influence on the rupture mode of unidirectional CFRPs, providing a numerical model of this influence. Rao et al. [14] conducted experimental and numerical investigations to provide a better understanding of the machining forces and workpiece damage caused by the orthogonal machining of unidirectional-CFRP and unidirectional-GFRP.

Numerical modeling of CFRP machining processes is presented as a supporting tool for the analysis of the influence of different variables, which are difficult to study experimentally, on the wear behavior of tools and on the damage found on workpieces due to machining processes. At the same time, the high costs associated with experimental tests are reduced. However, many numerical models found in the literature have been developed to simulate machining processes, which involve rotary cutting movements, such as turning [17,18] or drilling [19]. In these cases, it is difficult to obtain experimental information about the effect of the orientation of the fibers on the tool and on the workpiece, so the differences between the experimental test and the conditions considered in the model definition reduce the precision of the model. In general, 2D models have reduced cutting lengths, corresponding to a linear cutting movement in one single cutting pass [20,21,22,23]. Wang et al. [24] developed a numerical model, where the effect of the cutting speed (88–309 m/min), feed (0.1–0.45 mm) and fiber orientation (0–170°) in the orthogonal cutting of a rectangular unidirectional carbon fiber-reinforced polymer (unidirectional-CFRP) on workpiece damage was evaluated. The proposed methodology allowed for the analysis of these effects in approximately and extremely short cutting times per pass. The machined surface and internal integrity of the laminates was excellent, when the fiber orientation was 90° and 0°. Increasing the feed causes higher machining forces and more damage to the CFRP, and it was also observed that, as the cutting speed increased, the force and damage decreased.

In addition to the numerical model, the results obtained through orthogonal tests can be very useful for producing a better formulation of an analytical model, such as that of Sahraie Jahromi et al. [25], who developed an analytical model to predict the machining forces for the orthogonal machining of unidirectional polymer–matrix composites.

Experimental studies on the orthogonal cutting of composite materials allow for the analysis of the effect of the fiber orientation, tool geometry, chip formation and damage of laminates. Wang et al. [26] studied chip formation, workpiece damage and machining forces during the orthogonal cutting of unidirectional and multidirectional graphite epoxy composites. Li et al. [27] studied the chip removal process using a tungsten carbide tool in unidirectional CFRP. The main factors that affect the chip formation are the orientation of the fibers and the feed. A significant effect of contact pressure was shown in relation to different fiber orientations, as well as a less significant feed and rake angle effect. Voss et al. [28] performed a study to analyze the influence of tool geometry, fiber orientation and cutting parameters on the surface quality in machining CFRP. The increase of the clearance angle was found to reduce the contact area and therefore reduce the machining forces. Additionally, increasing the rake angle was found to improve the machined surface.

There is a lack of information on orthogonal cutting at a high cutting speed with linear displacement, because researchers mostly use modified milling machines, in which the cutter is fixed in the spindle of the milling machine, and the cutting speed is provided to the table [25,29]. Studies can also be found, in which a modified grinding machine is used to perform orthogonal cutting or turning machines to reach a higher cutting speed [17,18,24,30]. In Table 1, the cutting conditions used in previous publications, relative to the orthogonal cutting test and the used equipment, are summarized. This work focused on the experimental study of orthogonal cutting, with a linear movement at a high cutting speed, of a multidirectional CFRP laminate to analyze the influence of tool geometry and cutting parameters on material damage. The surface quality was determined with an optical microscope to analyze the damage, and the contact forces were measured during the orthogonal cutting.

## 2. Experimental Set-Up

In this section, the experimental equipment, including the materials, tools and measurement systems used in the machining tests, is briefly described.

### 2.1. Workpiece Material

The workpiece is a rectangular plate (100 mm × 20 mm × 2.2 mm), made of carbon fibers embedded in an epoxy matrix (IM7 MTM-45-1). A laminate of 16 layers of unidirectional CFRPs is oriented according to a sequence of [±45°/90°/0°] 2 s. The mechanical properties of the laminate are provided by the company, “Advanced Composite Group”, and are summarized in Table 2.

### 2.2. Tool Geometry

Two different types of carbide cutting tools are tested in the orthogonal cutting tests. These tools allow for a comparison of the effect of the cutting edge preparation in cutting processes with a small feed and the effect of the rake angle with a high feed on the cutting forces and workpiece damage. The real geometry of the cutting edge preparation of both tools is accurately determined by measurement using images obtained by scanning electron microscopy (SEM, Philips XL-30, (Philips, Amsterdam, Netherlands)).

**Figure 1:**
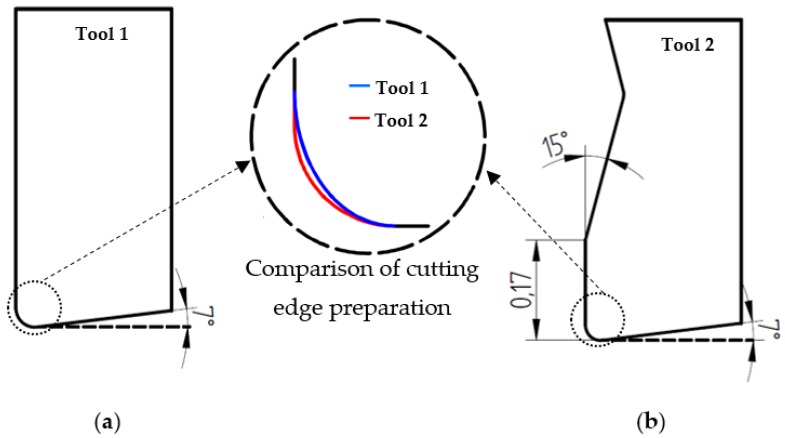
Details of the cutting edge geometry: (**a**) Tool 1 (uncoated-rounded); (**b**) Tool 2 (TiN coated-chamfered).

1. Tool 1 (provided by the manufacturer, Sandvik (Sandviken, Sweden): Uncoated cutting tool with a clearance angle of 7° and a rake angle of 0°. The cutting edge preparation is rounded with elliptical geometry, with a major axis of a radius of approx. 40 µm (in the direction of the rake surface) and a minor axis of a radius of approx. 30 µm (see details in Figure 1a). The cutting material grade is designed to be H13A.
2. Tool 2 (provided by the manufacturer, Seco (Fagersta, Sweden): Coated cutting tool (TiN) (designed material grade TS2000) with a clearance angle of 7°, a rake angle of 15°, and a chamfered-rounded cutting edge preparation. The chamfer width is 0.17 mm and the rounded honing radius is approx. 30 µm (see details in Figure 1b). The cutting edge preparation is designed to be F2.

Based on the results observed in the literature by other research groups in CFRP machining processes, the tribological effect of similar coating (present in tool 2) is related to slight reductions in machining force components [32]. However, it is taken into account for the analysis of the experimental results.

A wide range of cutting parameters is selected for the experimental test: Cutting speeds of 1, 50 and 200 m/min; and feeds of 0.05 mm, 0.1 mm and 0.2 mm. The cutting speed of 1 m/min is much lower than that used in industrial machining processes and has been considered in these tests as a parameter mainly to allow for comparison with other carbon fiber machining studies [13,14]. A maximum cutting speed of 200 m/min is selected due to the limitation of the used machine. However, it is enough to reach common industrial cutting speeds as the ones used in drilling processes. Férnandez-Pérez. et al. [33] used cutting speeds between 50 and 60 m/min in drilling processes. The used in this work cutting parameters are summarized in Table 3. The orthogonal cutting tests are performed under dry conditions to satisfy the industrial requirements [34].

### 2.3. Cutting Test Device with a Linear Desplacement

**Figure 2:**
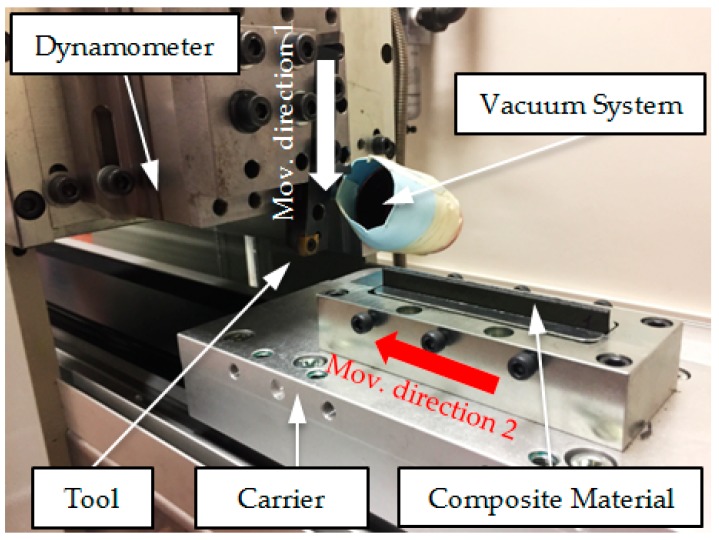
Implemented experimental set-up. Movement direction 1 is the feed control movement; movement direction 2 is the direction of the movement of the workpiece.

Experimental tests are carried out in an experimental system, designed for performing cutting tests with a linear movement. This device was developed by the authors to achieve a wide range of cutting speed. The workpiece is mounted on a carrier, which moves along toothed belt axes, according to a recirculating ball bearing guide (EGC-185.1500-TB-KF-GK) that is moved by a FESTO servo motor (EMMS-AS-140-L-HV-RMB). A support board was manufactured to place the tool holder and the cutting tool. The movement of the workpiece in the machine is controlled by a FESTO controller (CMMP-AS-C10-11A-P3-M0) connected to a computer. The chip thickness is controlled using the displacement of the tool with every cut and a dial gauge. The cutting test machine is generated using a dynamometer Kistler Model 9257B to measure the machining force components. The results of the experimental test are shown in Figure 2.

A Nilfisk S2B industrial vacuum system, with 2 kW of power, is attached to the machine to remove the powder-like chip particles generated during the machining of CFRP. The hard, broken fibers that separate from the laminate are harmful to human health when inhaled, so it is important to remove them for safety reasons. The laminates are inspected using an Optika SZR optical microscope (Optika, Ponteranica, Italy) to estimate the damage to different surfaces of, and measure the burr in, the CFRP laminate.

## 3. Results and Discussion

The experimental results are evaluated in terms of the machining forces, which are measured during the orthogonal cutting, and surface integrity.

**Figure 3:**
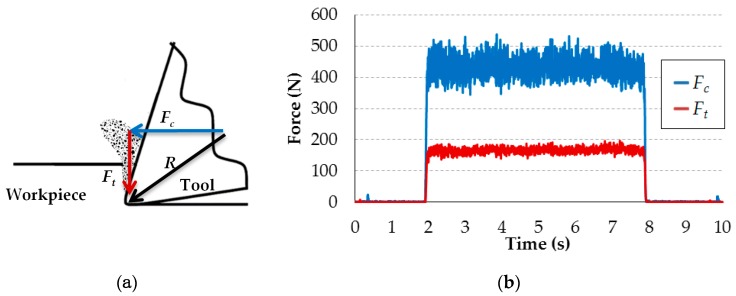
(**a**) Scheme of the machining force components; (**b**) Example of machining force evolution during a test (*v_c_* = 1 m/min; *f* = 0.2 mm).

The two components of the machining force associated with orthogonal cutting are measured: cutting force (*F_c_*) and thrust force (*F_t_*). *F_c_* is parallel to the cutting movement direction, and *F_t_* is perpendicular to the cutting movement. This is illustrated in Figure 3a. To confirm that during the tests, there is no significant tool wear, the cutting edges are inspected after each test by optical microscopy, and all tests are repeated twice, verifying that the results are repeatable, with differences in the force values below 3%. When the tool inspection or force values indicated that the tool wear is significant, the tool is replaced with a new one. Eight cutting edges are used during the tests to ensure that no significant tool wear is produced. One example of the evolution of the cutting forces during a test is shown in Figure 3b. Constant values of the machining forces are obtained during each cutting pass.

The surface integrity of the machined material is analyzed by optical microscopy.

### 3.1. Cutting Force

**Figure 4:**
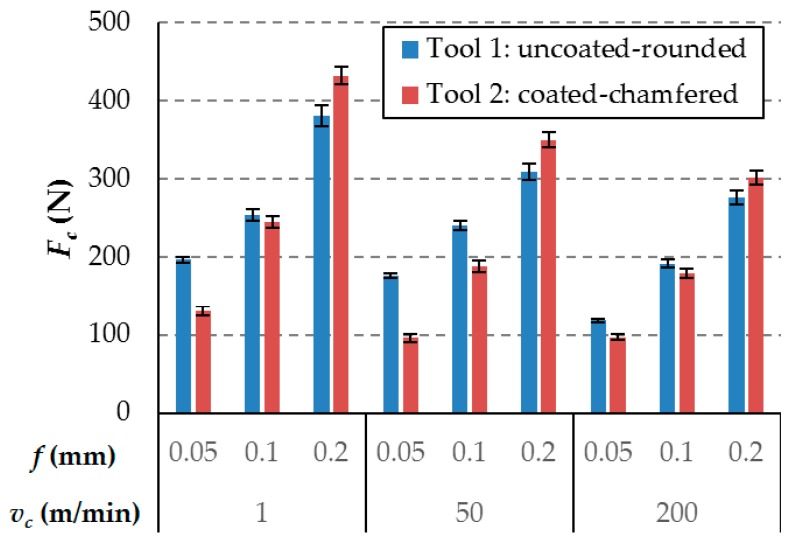
Cutting force component (*F_c_*) for all cutting conditions.

Figure 4 shows the component of the cutting force for all of the cutting conditions and cutting tools. It is observed that, for an undeformed chip thickness of 0.05 mm, tool 1 had higher cutting forces than tool 2. For feeds of 0.1 mm, the effect is less clear: the cutting force is higher for tool 1 with a cutting speed 50 m / min, but no significant variations are observed for the cutting speeds of 1 and 200 m / min. However, the cutting force values are lower for tool 1 with a feed of 0.2 mm. Therefore, the increase in the cutting force with the increase of the feed is clearly greater for tool 2, compared to tool 1, under all tested conditions. The area of the rake surface of tool 2, with the chamfered edge, has a highly positive rake angle (15°), which would have reduced the cutting force with a higher chip thickness. However, this geometric characteristic of tool 2 did not have an appreciable effect under the tested cutting conditions. Because the machined material exhibits an extremely fragile behavior during the chip formation process, the contact length between the chip and rake surface is similar to the value of the feeds. Therefore, even in the cutting tests with a higher feed, the entire working area of tool 2 practically corresponds to the chamfered edge area, which has the same rake angle as tool 1 (0°).

An increase in the feed increases the influence of the chip–tool contact on the force values obtained. Therefore, a possible tribological effect of the tool coating on this contact also increases. However, under the conditions tested, the TiN coating of tool 2, which is generally associated with reduced chip–tool friction forces, does not have an appreciable effect.

Therefore, the differences indicated in the Fc values are related mainly to geometrical differences in the rounding cutting edge. It should be noted that the tool manufacturers, Seco and Sandvik, indicate identical cutting edge rounding values for the two tools tested. However, as indicated in Section 2.2, tool 1 does have an elliptical sharpening geometry, with a radius greater than 40 microns (approx. 30% greater than the rounding radius of tool 2). Therefore, during machining, the thickness of the material affected by the rounded zone of the cutting edge (zone with an especially negative cutting geometry) is higher for tool 1. However, with a greater feed (0.2 mm), the Fc of tool 2 is slightly higher. It is difficult to determine, with certainty, the causes of this last observation, although it is expected that the configuration of the material, with fibers of different orientations, will have a significant influence, resulting in a strong cohesion of all of the materials affected by the cutting process. This cohesion means that, in tests with a feed of 0.2 mm, the machining efforts, corresponding to the material furthest from the cutting edge (upper part of the 0.2 mm layer), affect the machining in the rounded area of the edge and therefore the influence of the different geometries of the cutting edges of tools 1 and 2.

In a previous work [35] on cutting tools from the Seco and Sandvik tool manufacturers, it was observed that the real geometry of the cutting edge preparation and the criteria for quantifying the rounding radius applied by these tool manufacturers are significantly different.

The value of the cutting force varies within a range of 90 N to 440 N. Increasing the cutting speed produces lower levels of cutting force, this is because at high cutting speeds the cutting area reaches higher temperatures, which reduces the resistance of the matrix and therefore the resistance of the composite. This reduction in the cutting forces with the cutting speed was also previously observed by other authors at a lower cutting speed [11]. The growth of the cutting force component, between 5% and 40%, is observed with the reduction of the cutting speed. This parameter is less relevant than the chip thickness, as is shown in the ANOVA results in Table 3. The analysis indicates that all of the factors are relevant, because all of the estimated *p*-values are lower than 0.05. In the case of Tool 2, the influence of the cutting speed is less relevant. However, it is not negligible.

Higher values of the feed lead to increases in the cutting force component. It is observed that lower feed reduces the cutting force component for the uncoated rounded tool from 72% to 40%, and for the coated-chamfered-rounded tool, it reduces it from 57% to 22%. As indicated previously, these differences are related mainly to the geometrical differences in the rounding cutting edge. In Table 4, this factor presents a high contribution to the force, reaching a 90% in the case of the second tool. This means, that, for the same cutting speed, small variations in the undeformed chip thickness involve high differences in the cutting force component. An increase in the feed increases the section of machined material and therefore the number of fibers and matrix quantity affected by the machining process, as indicated above.

**Figure 5:**
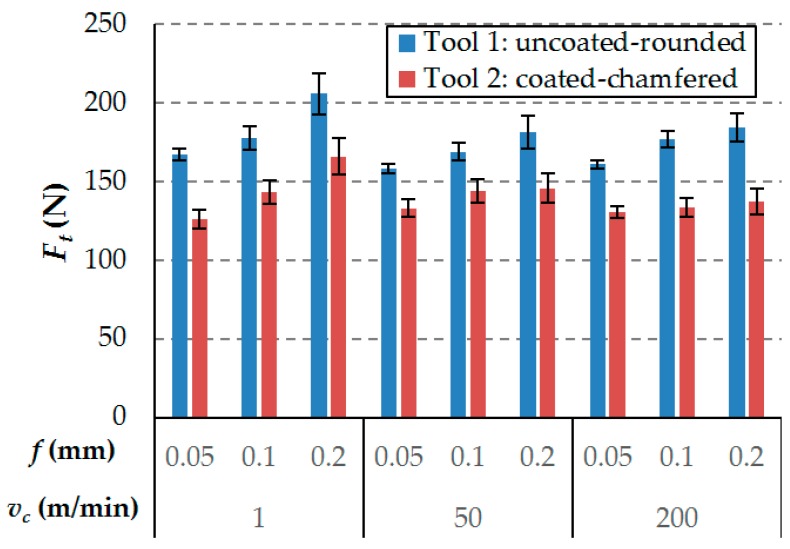
Thrust force component (*F_t_*) for all cutting conditions.

Regarding the thrust force component, it is observed that the uncoated rounded cutting tool (Tool 1) produces a higher thrust force, which increases the compressive loads in the laminate. This fact, illustrated in Figure 5, generates instabilities that could cause delamination damage, as will be discussed in the next section. The incrementation of the thrust force is more pronounced with high levels of feed for Tool 1. Considering the conclusion obtained from the previous analysis of Fc, and taking into account that both tools have the same clearance angle, the variations of Ft are mainly related to the differences in geometry and rounding cutting edge preparation radius of both tools described above plus the effect of the coating that helps to slightly decrease the thrust force [32].

Furthermore, moderate variations with the cutting parameters of the thrust force component were observed during the experimental tests, with values between 125 N and 205 N. It should be emphasized that, for the 2 tested tool geometries, feed has less effect on the thrust force than on the cutting force, these results agree with other author results [36]. As the feed increases the cutting force increases mainly because the undeformed chip cross section increases, however, as it was previously explained by Wang et al. [37]. As the tool pass through the specimen material part of the material is pushed down under the tool tip, the material that is pushed down has an important impact on the thrust force, however, since this phenom takes place only in the tip of the tool and considering that the rake angle of the studied tool, this effect shouldn’t vary much with the feed and it is why the feed has less impact on the thrust force than on the cutting force. However, according to the ANOVA results (see Table 5), feed has significant effect on this force. Therefore, high levels of feed led to a higher Fc/Ft ratio. Comparing the feed of 0.05 mm and 0.2 mm, an increment of the thrust force component between 5% and 24% was observed.

In contrast to the discussion of the cutting force, the thrust force component does not show a clear dependence on the cutting speed, as can be observed in Table 5, where the analysis of variance indicates that this parameter is not significant (*p*-value > 0.05).

### 3.2. Surface Integrity

**Figure 6:**
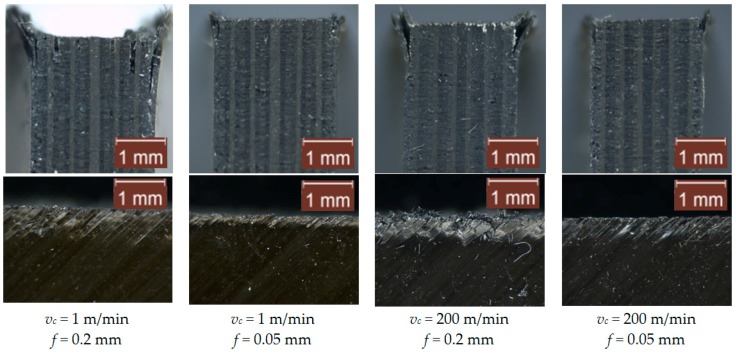
Images of the perpendicular surfaces of the machined surface of Carbon Fiber-Reinforced Plastics (CFRP) using the uncoated-rounded tool (tool 1). Note: The images at the top correspond to the frontal view of the specimen in the entrance plane. The images at the bottom correspond to the side view in the middle zone of the specimen.

**Figure 7:**
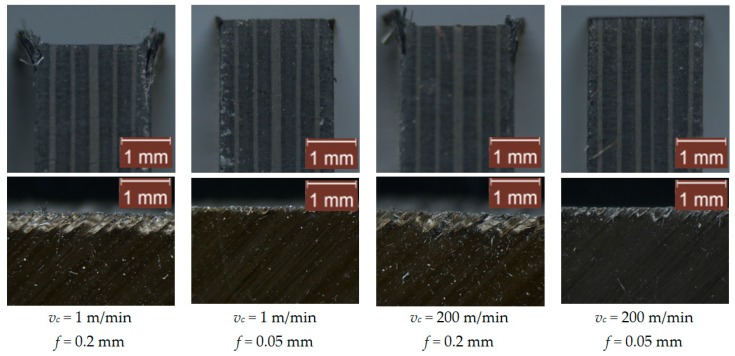
Images of the perpendicular surfaces of the machined surface of CFRP using the coated-chamfered tool (tool 2). Note: The images at the top correspond to the frontal view of the specimen in the entrance plane. The images at the bottom correspond to the side view in the middle zone of the specimen.

After observing the machined surfaces with a microscope, the main problems observed are the delamination damage caused by the machining of the laminates and the spalling generated in the first and the lasts plies of the laminate. Both damages are generated mainly by the load instabilities due to the compressive loads (thrust force component) on the machining, which produce interlaminar delamination. Interlaminar delamination is far more critical in the external plies, where there is no backup laminate, and this effect was already highlighted and described by Xu et al. [38]. Figure 6; Figure 7 show images of the tested materials, corresponding to 2 perpendicular planes of the machined surface for the tests at cutting speeds of 1 m/min and 200 m/min, and feeds of 0.05 mm and 0.2 mm, for the two tested cutting tools. For all images, the machined surface in the test corresponds to the top edge of the material.

In general, the surface damage is slightly higher in the tests with tool 1. This result is consistent with the higher thrust force (Figure 5) obtained for this tool. The best surface integrity results are found with a lower feed (0.05 mm), which is related to the lowest thrust force component. Increasing the feed involves elevating the delamination damage, and the first ply (45°) incurred the most damage. It should be noted that delamination occurs on the external plies, because there is no support structure. This problem could be reduced using lateral supports. It should be noted that increasing the cutting speed reduces the delamination damage in the laminates. However, the spalling of the fibers is not reduced. Nevertheless, the effect of the cutting speed on the surface integrity is less significant than the effect of the feed. As shown in the ANOVA results, presented in the previous section, the effect of the feed on the thrust force is larger than the one observed for the cutting speed.

**Figure 8:**
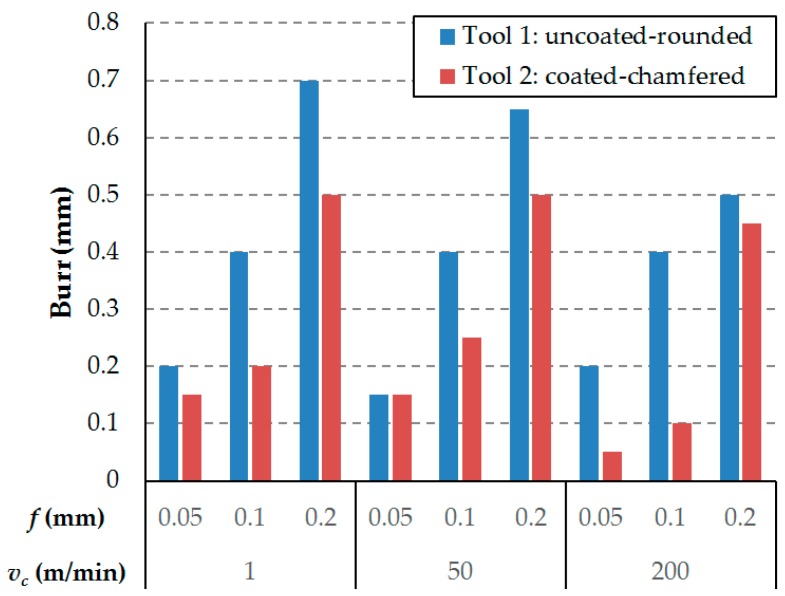
Burr length after a cutting pass.

The delamination observed in the material is related to the burr present in the external plies. The length of the burr could be a parameter that quantifies the damage caused by machining composite laminates. The length from the machined surface to the end of the burr is measured using an optical microscope. Figure 8 shows the results of the burr length measurement during the observation of the samples. It is observed that larger feeds lead to larger burr length. This is in line with the force measurements and damage inspection results, and the influence of the feed parameter is still more significant than that of the cutting speed.

The influence of the cutting tool, the feed and the cutting speed on the surface integrity is analogous to that observed in the experimental results for the thrust force. Therefore, as explained in previous paragraphs, compression forces (thrust forces) create instabilities which lead to less effected cut in the laminates that do not present backup supports as the external ones, where the laminates tend to bend with the action of the tool. Hence, higher thrust forces correspond to greater surface damage due to machining.

## 4. Conclusions

The orthogonal cutting of CFRP, with a linear movement and a wide range of cutting speeds and feeds, has been analyzed in this paper using two different cutting tools. For this purpose, an experimental device, based on the linear movement of a carrier in toothed belt axes, with a servomotor, has been employed.

The main conclusions obtained in the experimental work are summarized below:

1. The developed device is suitable for cutting tests, with a linear displacement and a wide range of cutting parameters.
2. The possibility of reaching a high cutting speed during orthogonal cutting allows the author to confirm the tendencies found by other authors at a lower cutting speed. Increasing the feed increases the cutting force and the thrust force components. This causes an increase in the compressive loads and instabilities, which could damage the laminate. The increase of the cutting speed lead to a reduction of the cutting force, but it does not have a significant effect on the thrust force for the studied range of cutting speeds.
3. The thrust force and the cutting force variations are mainly related to differences in the feed. Also, the cutting-edge preparation of the tools has a significant effect in the machining force components.
4. Delamination is the main type of damage that the machined surfaces of the CFRP laminates presented. The delamination is noticeable in the external plies of the laminates. In addition, spalling of these plies is also produced.
5. The influence of the tool geometry, the feed and the cutting speed on the surface integrity is analogous to that observed in relation to the thrust force. Therefore, a higher feed is related to higher forces and also more surface damage due to machining. Additionally, a lower but significant effect on surface integrity is observed when comparing tool types and cutting speeds.

## Figures and Tables

**Table 1:** Cutting conditions and equipment review.

Author	Format	Cutting Speed (m/min)	Feed (mm)	Cutting Machine
Rao et al. [14]	Plate	0.5 m/min	0.1, 0.15 and 0.2 mm	CNC machine (no description)
Wang et al. [15]	Plate	0.3 m/min	0.001–0.05 mm	CNC machine (no description)
Seeholzer [18]	Cylindrical	90 m/min	0.03 mm	Modified CNC lathe (Okuma LB15-II)
Voss et al. [17]	Rings	20 m/min-500 m/min	0.03 mm	-
Wang et al. [24]	Plate	88.4–309.5 m/min	0.1–0.45 mm	Turning special device
Bhatnagar et al. [31]	Plate	0.5 m/min	0.1, 0.2, and 0.3 mm	Modified CNC milling
Nayak et al. [29]	Plate	0.5 m/min	0.1, 0.2, and 0.3 mm	Modified CNC milling
Li et al. [27]	Plate	0.5 m/min	0.1, 0.2 and 0.5 mm	Modified CNC milling (JOHNFORD VMC-850)
Saheaie et al. [25]	Plate	0.354 m/min	0.1	Modified CNC milling
An et al. [30]	Plate	200 m/min	0.02 mm	Modified surface grinder (KENT-KGS-1020AH)
Wang et al. [26]	Plate	4–14 m/min	0.127–0.381 mm	Rockford Planer-Shaper, equipped with a hydraulic table

**Table 2:** Mechanical properties of the composite material.

Properties	Value
Density *ρ* (Kg/m^3^)	1534
Young’s Modulus *E*_1_ (GPa)	173
Young’s Modulus *E*_2_ = *E*_3_ (GPa)	7.36
Major Poisson ratio *ν*_21_	0.33
In-Plane Shear Modulus *G*_12_ (GPa)	3.89

**Table 3:** Cutting conditions of the experimental test.

Cutting Parameters	
Cutting speed (*v_c_*)	1, 50 and 200 m/min
Feed (f)	0.05, 0.1 and 0.2 mm

**Table 4:** ANOVA analysis of the cutting force component (*F_c_*). DF: degrees of freedom; Significant parameters have an F-Ratio > F (*α* = 5%) = 6.94 and a *p*-value < 0.05.

Tool	Factor	Sum of Squares	DF	Mean Square	F-Ratio	*p*-Value	Contribution
Tool 1	*v_c_*	10076.90	2	5038.45	18.69	0.009344908	20.70%
	*f*	38093.01	2	19046.50	70.65	0.000757875	78.2%
	Error	1078.37	4	269.59			1.1%
	Total	49248.28	8				
Tool 2	*v_c_*	9605.56	2	4802.78	7.95	0.040403932	8.9%
	*f*	97624.68	2	48812.34	80.780	0.00058348	90.0%
	Error	2416.53	4	604.13			1.1%
	Total	109646.77	8				

**Table 5:** ANOVA analysis of the thrust force component (*F_c_*). DF: degrees of freedom; Significant parameters have an F-Ratio > F (*α* = 5%) = 6.94 and a *p*-value < 0.05.

Tool	Factor	Sum of Squares	DF	Mean Square	F-Ratio	*p*-Value	Contribution
Tool 1	*v_c_*	317.64	2	158.82	4.68	0.089587	19.8%
	*f*	1220.28	2	610.14	17.99	0.010013	76.0%
	Error	135.69	4	33.92			4.2%
	Total	1673.61	8	802.88			
Tool 2	*v_c_*	192.52	2	96.26	1.17	0.398281	20.6%
	*f*	578.82	2	289.41	3.51	0.131519	61.8%
	Error	329.35	4	82.34			17.6%
	Total	1100.70	8	468.01			
